# Unraveling predicted immunomodulatory effects of novel cancer-associated non-coding RNAs

**DOI:** 10.1186/2051-1426-3-S2-P396

**Published:** 2015-11-04

**Authors:** Antoine J Tanne, Luciana R Muniz, David T Ting, Alexander V Solovyov, Arnold Levine, Benjamin Greenbaum, Nina Bhardwaj

**Affiliations:** 1 Icahn School of Medicine at Mt Sinai, New York, NY, USA; 2 Massachusetts General Hospital, Charlestown, MA, USA; 3 Institute for Advanced Study, Princeton, MA, USA

## 

Cellular non-coding RNA (ncRNA) transcription and regulation has only recently been systematically investigated. Studies have demonstrated abundant transcription of a set of ncRNAs preferentially within tumors as opposed to normal tissue. In some cases these tumor-associated ncRNAs also associate with inflammatory cytokine production suggesting potential immunostimulatory qualities. Using a novel approach from statistical physics we quantify global transcriptome-wide motif usage for the first time in human and murine ncRNAs determining that most have motif usage consistent with the coding genome. However an outlier subset of tumor-associated ncRNAs typically of recent evolutionary origin has motif usage more associated with pathogen RNA. Here we show that a human repeat enhances immunostimulatory motifs CpG dinucleotides in AU-rich contexts which most of the human genome and human adapted viruses have evolved to avoid. We demonstrate that a key subset of these ncRNA function as immunostimulatory “self-agonists” and directly activate cells of the mononuclear phagocytic system to produce pro-inflammatory cytokines. These ncRNAs arise from endogenous repetitive elements that are normally silenced, yet are often very highly expressed in cancers. We conclude that the innate response in tumors partially originates from direct interaction of immunogenic ncRNAs expressed in cancer cells with innate pattern recognition receptors and thereby assign a new danger-associated function to a set of dark matter repetitive elements, potentially reconciling several observations concerning the role of ncRNA expression in cancers. We consider the possible roles they may play in the tumor microenvironment.

